# Interhemispheric Resting-State Functional Connectivity Predicts Severity of Idiopathic Normal Pressure Hydrocephalus

**DOI:** 10.3389/fnins.2017.00470

**Published:** 2017-09-01

**Authors:** Yousuke Ogata, Akihiko Ozaki, Miho Ota, Yurie Oka, Namiko Nishida, Hayato Tabu, Noriko Sato, Takashi Hanakawa

**Affiliations:** ^1^Department of Advanced Neuroimaging, Integrative Brain Imaging Center, National Center of Neurology and Psychiatry Kodaira, Japan; ^2^Biointerfaces Unit, Institute of Innovative Research, Tokyo Institute of Technology Yokohama, Japan; ^3^Department of Functional Brain Research, National Institute of Neuroscience, National Center of Neurology and Psychiatry Kodaira, Japan; ^4^Social Welfare Organization Saiseikai Imperial Gift Foundation, Inc., Osaka Saiseikai Nakatsu Hospital Osaka, Japan; ^5^Department of Mental Disorder Research, National Institute of Neuroscience, National Center of Neurology and Psychiatry Kodaira, Japan; ^6^Kitano Hospital, Tazuke Kofukai Medical Research Institute Osaka, Japan; ^7^Department of Radiology, National Center of Neurology and Psychiatry Hospital Kodaira, Japan; ^8^Department of Clinical Neuroimaging, Integrative Brain Imaging Center, National Center of Neurology and Psychiatry Kodaira, Japan

**Keywords:** resting-state functional connectivity MRI, idiopathic normal pressure hydrocephalus, functional connectivity, supervised machine learning, support-vector machine, interhemispheric connectivity

## Abstract

Idiopathic normal pressure hydrocephalus (iNPH) is characterized by a clinical triad (gait disturbance, dementia, and urinary incontinence), and by radiological findings of enlarged ventricles reflecting disturbance of central spinal fluid circulation. A diagnosis of iNPH is sometimes challenging, and the pathophysiological mechanisms underlying the clinical symptoms of iNPH remain largely unknown. Here, we used an emerging MRI technique, resting-state functional connectivity MRI (rsfcMRI), to develop a subsidiary diagnostic technique and to explore the underlying pathophysiological mechanisms of iNPH. rsfcMRI data were obtained from 11 patients with iNPH and 11 age-matched healthy volunteers, yielding rsfcMRI-derived functional connectivity (FC) from both groups. A linear support vector machine classifier was trained to distinguish the patterns of FCs of the patients with iNPH from those of the healthy volunteers. After dimensional reduction, the support vector machine successfully classified the two groups with an accuracy of 80%. Moreover, we found that rsfcMRI-derived FC carried information to predict the severity of the triad in iNPH. FCs relevant to the classification of severity were mainly based on interhemispheric connectivity, suggesting that disruption of the corpus callosum fibers due to ventricular enlargement may explain the triad of iNPH. The present results support the usefulness of rsfcMRI as a tool to understand pathophysiology of iNPH, and also to help with its clinical diagnosis.

## Introduction

Idiopathic normal pressure hydrocephalus (iNPH) is a cryptogenic disorder characterized clinically by a triad (gait disturbance, cognitive impairment and urinary incontinence), and radiologically by enlarged cerebral ventricles because of non-obstructive disturbance of cerebrospinal fluid (CSF) circulation (Ishikawa, 2004). The diagnosis of iNPH is important, because it is one of the few treatable dementias; namely, the clinical symptoms can be often relieved by a shunt operation. Although typical iNPH shows characteristic neuroradiological findings (Kitagaki et al., 1998; Ishikawa et al., 2008; Mori et al., 2012), the diagnosis of iNPH remains challenging in atypical cases, because no functional biomarkers of iNPH have been established (Leinonen et al., 2011; Jingami et al., 2015). The clinical guidelines of iNPH in Japan recommend a “tap test” involving the drainage of a small amount of CSF (30–50 ml) by a lumbar puncture to predict the effectiveness of a shunt operation. However, the tap test has limitations: it is an invasive procedure and carries some risks of adverse events, such as headache. Additionally, although a positive tap test result has a high predictive value for shunt operation outcomes, a negative result has a low predictive value because of a high false negative rate (Sakakibara et al., 2012). Thus, it is important to develop non-invasive and reliable diagnostic methods for iNPH. In particular, a functional neuroimaging method would not only help with a diagnosis of iNPH, but would also provide insight into the pathophysiological mechanisms of iNPH.

Recently, resting-state functional connectivity MRI (rsfcMRI) has drawn attention as a tool to help with a clinical diagnosis and the evaluation of neuropsychiatric disease. For rsfcMRI, participants are not required to perform demanding cognitive tasks, instead they only have to lie quietly in an MRI scanner. This property is advantageous for the application of rsfcMRI to patients with dementia, who might have difficulty in performing cognitive tasks. In fact, rsfcMRI has recently been applied to neuropsychiatric disorders (Greicius et al., 2004; Damoiseaux et al., 2006; Zhang et al., 2010; Khoo et al., 2015; Takamura and Hanakawa, 2017). In particular, Khoo et al. reported that resting-state functional connectivity (RSFC) was reduced in the default mode network (DMN) in iNPH patients, and that the reduction of the RSFC in DMN was positively correlated with symptoms of iNPH (Khoo et al., 2015). This pioneering study indicates that RSFCs may provide a useful biomarker for the assessment of iNPH. However, an alteration of RSFCs other than DMN remains unclear, because the analysis was limited to the DMN. Thus, it is important to test the contribution of multiple large-scale brain networks to the diagnosis of patients with iNPH. In this regard, an emerging method for a multivariate RSFC analysis is the application of machine-learning classification algorithms (Craddock et al., 2009; Arbabshirani et al., 2013). For example, Craddock and colleagues successfully applied support-vector machine (SVM) classification to identifying a pattern of RSFCs representing major depressive disorder (Craddock et al., 2009). A previous study (Arbabshirani et al., 2013) demonstrated that SVM with RSFC features could classify more accurately patients with neuropsychiatric disease than other linear classification methods. In addition, the SVM is particularly suitable to deal with the classification task with limited number of the training instances, and high feature dimensionality. This availability of SVM is suitable to clinical neuroimaging studies where typically just a limited number of dataset is available.

Recently, several researchers used SVM to examine the diagnostic, and prognostic potential of structural MRI, functional MRI, and rsfcMRI in various areas of neurological, and psychiatric disorders, including mild cognitive impairment, probable dementia of Alzheimer type, major depression, bipolar disorder, and schizophrenia (Chen et al., 2011; Costafreda et al., 2011; Orru et al., 2012; Wee et al., 2012; Zeng et al., 2012).

However, to our knowledge, machine-learning classification has not been applied to iNPH. A supervised machine-learning classification algorithm uses multivariate datasets such as whole-brain RSFC, and is able to classify the data into predetermined categories (i.e., iNPH and healthy). Moreover, a multivariate pattern analysis on RSFC has the potential to classify the severity of disease, like the iNPH grading scale (iNPH-GS).

In this study, we applied SVM to rsfcMRI data to test the hypothesis that differences in RSFCs might differentiate between iNPH patients, and healthy volunteers, and might also discriminate across the severity stages of iNPH symptoms.

## Methods

### Participants

Eleven patients with iNPH (iNPH group; 5 male, 6 female; average age ± *SD*: 78.2 ± 6.8 years) and 11 age-matched healthy controls (HC group; 4 male, 7 female; average age ± *SD*: 69.8 ± 13.6) participated in the study. The two groups did not significantly differ in terms of age (two-sample *t*-test, *p* = 0.07) or sex (chi-square test, *p* = 0.53). Nine out of 11 patients fulfilled the criteria for probable iNPH (Ishikawa, 2004; Yamashita et al., 2014): (1) age of 60 years or older; (2) presence of at least one of the classic triad (gait disturbance, cognitive impairment, and urinary incontinence); (3) ventricular dilation with Evans' index >.3; (4) normal CSF pressure and content; (5) exclusion of other neurological or non-neurological disorder; and (6) a positive CSF tap test. Two patients were diagnosed as possible iNPH because the result of a CSF tap test was equivocal. The general clinical status of the iNPH patients was assessed with the iNPH grading scale (iNPH-GS) (Kubo et al., 2008). The iNPH-GS grades the severity of three main categories of symptoms (cognitive impairment, gait disturbance, and urinary disturbance) with five levels ranging from 0 (normal) to 4 (severe). Additionally, cognitive impairment was assessed with the Mini-Mental State Examination (MMSE), and the Frontal Assessment Battery (FAB). From the HC group, only the MMSE was available. The score of the MMSE showed significant differences between the iNPH group (22.8 ± 4.3, mean ± *SD*) and the HC group (28.9 ± 1.0) [*T*_(20)_ = 3.93, *p* < 0.001]. The profiles and clinical information of the iNPH patients are summarized in Table 1.

**Table 1:** Profiles of the iNPH patients and healthy controls.

	**Age group**	**MMSE**	**FAB**	**iNPH-GS gait**	**iNPH-GS cognition**	**iNPH-GS urinary**	**CSF tap test**
iNPH1	80–85	17	10	3	3	0	Positive
iNPH2	80–85	25	11	2	1	1	Positive
iNPH3	70–75	22	10	2	2	0	Positive
iNPH4	70–75	16	9	3	3	3	Positive
iNPH5	76–80	19	6	3	2	2	Positive
iNPH6	70–75	28	13	2	2	2	Positive
iNPH7	80–85	25	6	2	2	0	Positive
iNPH8	66–70	29	13	1	2	0	Negative
iNPH9	76–80	25	17	1	1	0	Negative
iNPH10	76–80	25	16	2	2	4	Positive
iNPH11	86–90	20	12	2	3	2	Positive
iNPH (mean ±*SD*)	78.2 ± 6.8	22.8 ± 4.3	11.2 ± 3.5	2.1 ± 0.7	2.1 ± 0.7	1.3 ± 1.4	
Control (mean ±*SD*)	69.8 ± 13.6	28.9 ± 1.0	NA	NA	NA	NA	NA

*MMSE, Mini-Mental State Examination; FAB, Frontal Assessment Battery; iNPH-GS, idiopathic normal pressure hydrocephalus grading scale; NA, not available*.

The iNPH patients were enrolled at the Kitano Hospital and the healthy participants were enrolled at the National Center of Neurology and Psychiatry. All participants gave their informed consent prior to their inclusion in the study according to the study protocol approved by the institutional ethics committee.

### rsfcMRI acquisition

For rsfcMRI, the participants were scanned for 7 min with a 3T-MRI system (Achieva 3.0TX, Philips Medical Systems, Best, The Netherlands). The participants were asked to stay relaxed and to close their eyes, but not to fall asleep, in a comfortable position within the scanner. They were also instructed not to think about anything in particular, and not to imagine specific figures or scenes. RsfcMRI data were acquired with a T_2_^*^-weighted echo-planer imaging (EPI) sequence using the following parameters: repetition time (TR) = 3,000 ms, echo time (TE) = 30 ms, flip angle (FA) = 90°, 3-mm slice thickness with a 1-mm gap, voxel size = 1.875 × 1.875 × 3 mm, and 34 slices (descending order). In total, 120 volumes were acquired, and the first 4 volumes were discarded to exclude signal changes arising from T1 saturation effects.

### Extracting functional connectivity

Preprocessing of the rsfcMRI data was conducted with Statistical Parametric Mapping 8 (SPM8, Well-come Department of Imaging Neuroscience, London, UK) implemented on MATLAB (MATHWORKS, Inc., MA, USA). After slice-timing correction was applied to the EPI images, the images were realigned to the first image, using a rigid-body transformation for correcting head motion. Then, the images were spatially normalized to the EPI image template conforming to the Montreal Neurological Institute (MNI) space, using the SPM normalization algorithm. These normalized images were resampled to a 3 × 3 × 3-mm^3^ voxel size. The normalized EPI images were then spatially smoothed with an isotropic Gaussian kernel of 6-mm full width at half maximum. In addition, we computed gray matter, white matter and cerebrospinal fluid (CSF) images as binary masks for later use. High-quality three-dimensional anatomical images were available for the healthy controls, but not for the iNPH patients due to the limitation of scanning time. To avoid introducing a bias to the WM/CSF mask images between the groups, we directly segmented the normalized EPI images into GM, WM, and CSF images in both groups, by using a segmentation algorithm implemented in SPM8. The purpose of the segmentation here was to create masks for WM and CSF to compute signals to be regressed out from the data (denoising). This is recommended for the analysis of rsfMRI since signals from CSF and WM produce pseudo-correlations, resulting in non-zero mean and large standard deviations of the functional connectivity data (Whitfield-Gabrieli and Nieto-Castanon, 2012) For the quality check of our de-noising procedure using the EPI-derived WM, and CSF masks, we computed the degree to which false correlation was corrected by removing the signal from WM, and CSF. The QC results showed that the distribution of correlation coefficients was reasonably corrected to nearby zero mean and small standard deviation (before removal; average of mean = 0.18, average of standard deviation = 0.40, after removal; average of mean = 0.09, average of standard deviation = 0.21). Judging from this QC result, we believe that segmentation procedure has provided WM/CSF signals of reasonable quality to reduce the pseudo-correlation.

After these preprocessing steps, the CONN toolbox (Whitfield-Gabrieli and Nieto-Castanon, 2012) was used for conducting a region-of-interest (ROI)-to-ROI analysis to compute RSFC, indexing the degree of correlation between MRI signal time-courses in each pair of ROIs. For defining ROIs, 90 ROIs were defined according to the automated anatomical labeling (AAL) template implemented in the WFU Pickatlas (Tzourio-Mazoyer et al., 2002; Maldjian et al., 2003). First, we used standard AAL ROIs to compute FC. However, in this procedure, some ROIs overlapped the enlarged ventricles only in the iNPH group. To reduce the effects of the ventricle enlargement onto signals from ROIs, we next created modified AAL ROIs, by removing the intersection of the AAL, and the iNPH ventricle template. The iNPH ventricle template represented a map of voxels affected by enlarged ventricle in a group of iNPH patients compared with a group of healthy elderly subjects (Yamashita et al., 2010). We applied the same set of the modified AAL ROIs to the iNPH patients and healthy subjects. Because the results of SVM classification were essentially the same between the standard and modified AAL versions, we only described the results from the modified AAL approach for simplicity. For the removal of signals of no interest, signals correlated with six motion parameters from the realignment procedure and signals derived from the entire WM and CSF mask were regressed out in each participant, by using a general linear model-based multiple regression.

After that, a signal time-series averaged across voxels was extracted from each ROI. Next, all of the confound-removed time course data underwent band-pass filtering of 0.001–0.1 Hz. Then, the correlation coefficient of the BOLD signal time-course was computed between all the pairs of ROIs, and Fisher's z-transform was applied for each coefficient, yielding an FC matrix including all the ROI pairs in each participant.

### Classification analysis using support-vector machine

We tested whether RSFC information could classify each participant into the iNPH group or the HC group. To do so, we used a linear SVM, a machine-learning algorithm implemented in the e1071 machine-learning package of R version 3.1.3. SVM is a method for classifying data consisting of multi-dimensional features. SVM labels each data input by computing a hyperplane that best classifies the data in the feature space. Under the context of supervised machine learning, SVM identifies the closest data to a hyperplane as sample (or representative) data, and reconfigures the hyperplane to maximize the distance between the sample data with different labels. We used a z-transformed FC between each ROI pair as a feature and computed weights on the FC for successful classification of the iNPH and HC groups. We also tested if RSFC was able to predict the severity of iNPH patients (i.e., iNPH-GS). For the prediction of iNPH-GS, multi-class SVM with a one-against-all classification method was used. Because the iNPH-GS score of iNPH participants in the present cohort varied from 0 to 3 in the gait assessment, from 0 to 3 in the cognitive assessment, from 0 to 4 in the urinary assessment, these scores were set as a label for classification. Because of the limited number of participants, we included data from healthy volunteers for the training of SVM. Because iNPH-GS assessment was not available for the HC group, the label of these data was assumed to be 0 (normal). First, all the FC pairs were fed in to the SVM algorithm in both analyses as feature quantity. All of the features were scaled internally to zero mean and unit variance. The SVM model was constructed with parameters as follows: linear kernel function, C-classification algorithm, and cost factor = 1. Also, to avoid a bias arising from the asymmetry of labels, we defined class the weight vector as 1 divided by number of each labels (Chang and Lin, 2011). Second, we used a feature selection method with a *t*-test filter to reduce the feature dimension in view of the excess of features against the number of data samples (Craddock et al., 2009). For the *t*-test filter, we applied a two-sample *t*-test on the FC comparing between iNPH and HC, and then employed FC with a difference (*p*-value threshold = 0.05). To validate the SVM model for predicting a new data set, we employed a leave-one-out cross validation (LOOCV) procedure. In the LOOCV analysis, an SVM was trained with FC from a randomly selected 21 out of the 22 participants. After learning, the performance of the SVM classifier was tested to classify the remaining participant. After this procedure was repeated until the FC from every participant had become the test data, we calculated the classification accuracy as the number of correct labels divided by the number of LOOCV tests. To evaluate the specificity of classification accuracy for iNPH patients, we limited the LOOCV test round to the data derived from the iNPH patients in the iNPH-GS classification. A binomial test was used to determine the significance of the classification accuracy.

## Results

### Classification between iNPH and HC

**Figure 1:**
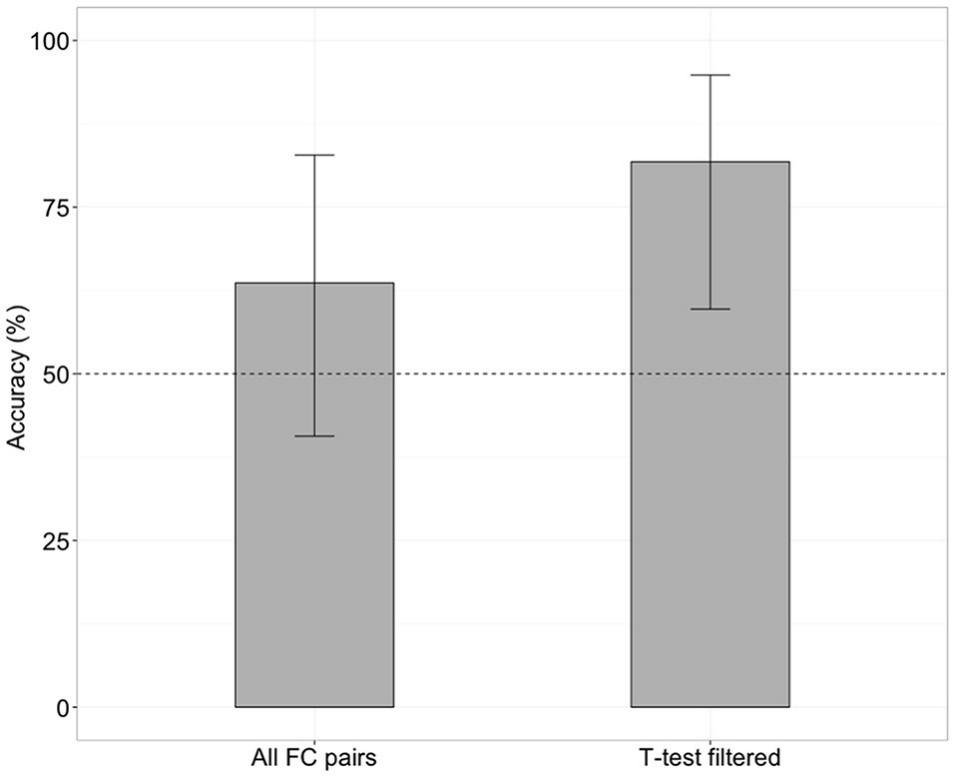
Classification accuracy without **(left)** and with **(right)** future selection. Error bars mean 95% confidence interval from a binomial test.

When the SVM was trained with all 4,005 pairs of the FC (no feature selection), the classifier was able to distinguish iNPH from HC with an accuracy of 63% (Figure 1), which was not significantly different from chance level (binominal test, 14 of 22 LOOCV tests, *p* = 0.28). However, when the SVM was trained with 476 features after the feature selection (*t*-test filtering), classification accuracy was 81% (Figure 1), which was significantly above chance level (18 of 22 tests, *p* < 0.01).

**Figure 2:**
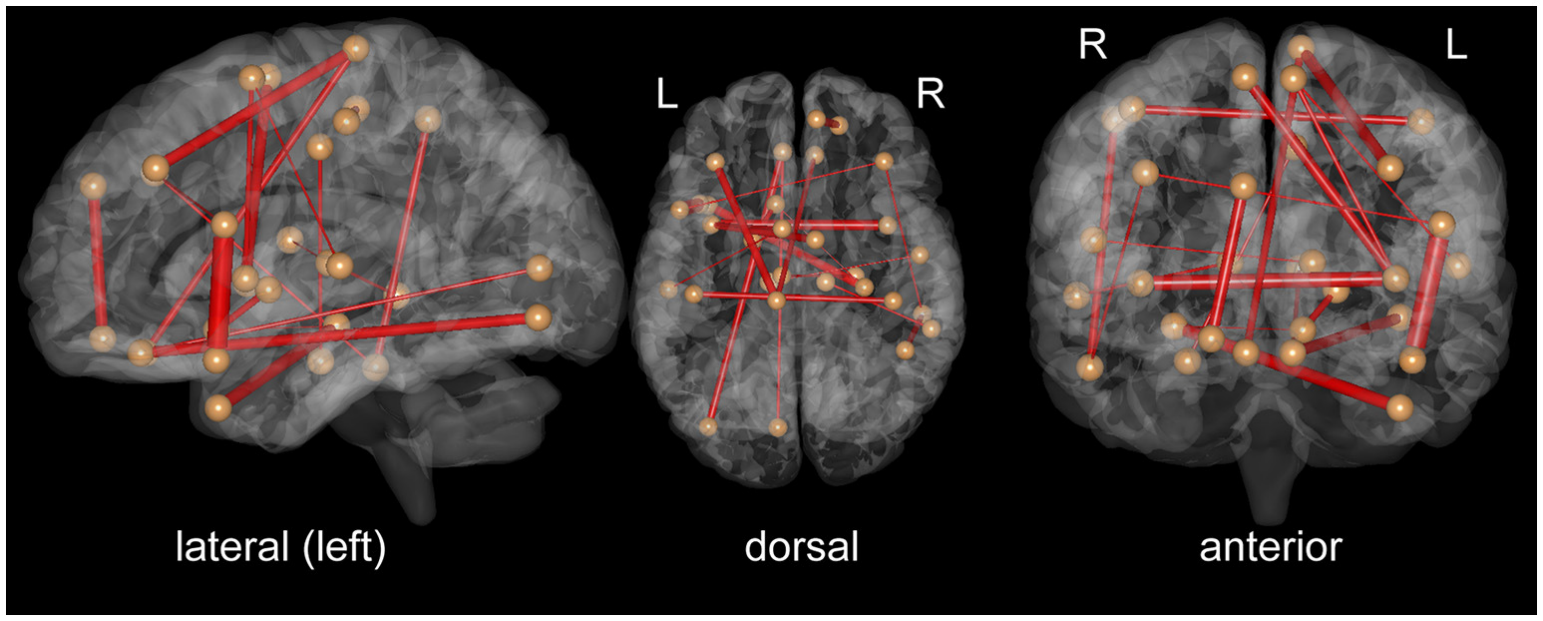
Top 20 functional connectivity pairs contributing to the classification between iNPH and HC groups in the views from the left **(left)**, top **(middle)**, front **(right)**. The spheres represent the barycenter of ROIs, and the lines connecting spheres stand for edges with high SVM weights listed in Table 2. The thickness of the lines represents relative SVM weights.

To clarify which FC was important for the classification, we assessed SVM weights, representing the degree of contribution of each FC to the classification. We found that relatively high weights were placed onto the FC between the left superior temporal pole and left inferior frontal operculum, the bilateral insula, the left middle temporal pole, and right hippocampus, the right superior medial frontal cortex, and left superior orbitofrontal cortex, and the left paracentral lobule, and left medial frontal cortex. Interhemispheric connectivity accounted for more than half (56%) of the FCs that contributed to the classification after the *t*-test filtering. Figure 2, Table 2 show the top 20 FC with high-level weights. These 20 weights accounted for 15.6% of the total weights.

**Table 2:** SVM weights contributing to the classification of iNPH and HC.

**Functional connectivity**	**Weight of SVM**
L superior temporal pole–L inferior frontal operculum	0.0072
R insula–L insula	0.0064
L middle temporal pole–R hippocampus	0.0061
R superior frontal gyrus, medial orbital–L superior frontal gyrus, orbital part	0.0059
L paracentral lobule–L medial frontal cortex	0.0059
L inferior occipital gyrus–L gyrus rectus	0.0058
L insula–R supplementary motor area	0.0058
R postcentral gyrus–L postcentral gyrus	0.0055
R inferior temporal gyrus–R inferior parietal gyrus	0.0054
L paracentral lobule–R gyrus rectus	0.0053
L pallidum–L olfactory cortex	0.0052
L insula–L supplementary motor area	0.0049
L calcarine fissure–L gyrus rectus	0.0048
R parahippocampal gyrus–L median cingulate gyrus	0.0047
R inferior temporal gyrus–R middle frontal gyrus	0.0046
R middle temporal gyrus–R thalamus	0.0046
L superior temporal gyrus–L supplementary motor area	0.0045
L inferior frontal gyrus, opercular part–R middle frontal gyrus	0.0045
L thalamus–R rolandic operculum	0.0044
R hippocampus–L olfactory cortex	0.0043

### Prediction of iNPH-GS

**Figure 3:**
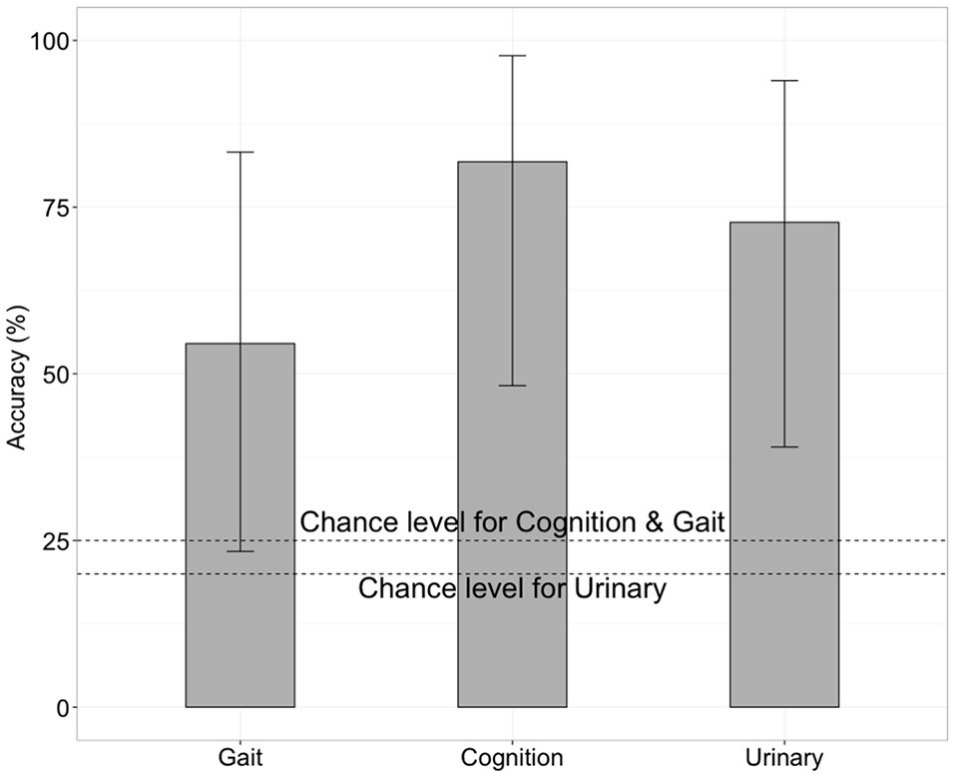
Classification accuracy for severity of iNPH symptoms when we limited the test round to the data derived from the iNPH patients, using functional connectivity as the classification feature. Error bars mean 95% confidence interval of binomial test.

We next tested whether the RSFCs contained information about the severity of iNPH-related symptoms. Specifically, we applied SVM with LOOCV to FCs to predict the level of gait disturbance, cognitive impairment and urinary incontinence as indexed by iNPH-GS. This was done after the feature selection by applying *t*-test filtering comparing the two groups as described above. We found that FC contained significant information concerning the severity of iNPH (Figure 3). Classification accuracy was significantly higher than chance when we limited the test round to the data derived from the iNPH patients: 81% for the iNPH-GS cognition (9 out of 11 tests, *p* < 0.05 by a binominal test), 55% for the iNPH-GS gait (6 out of 11 tests, *p* < 0.001), and 72% for the iNPH-GS urinary score (8 tests of 11 tests, *p* < 1.0 × 10^−7^).

**Figure 4:**
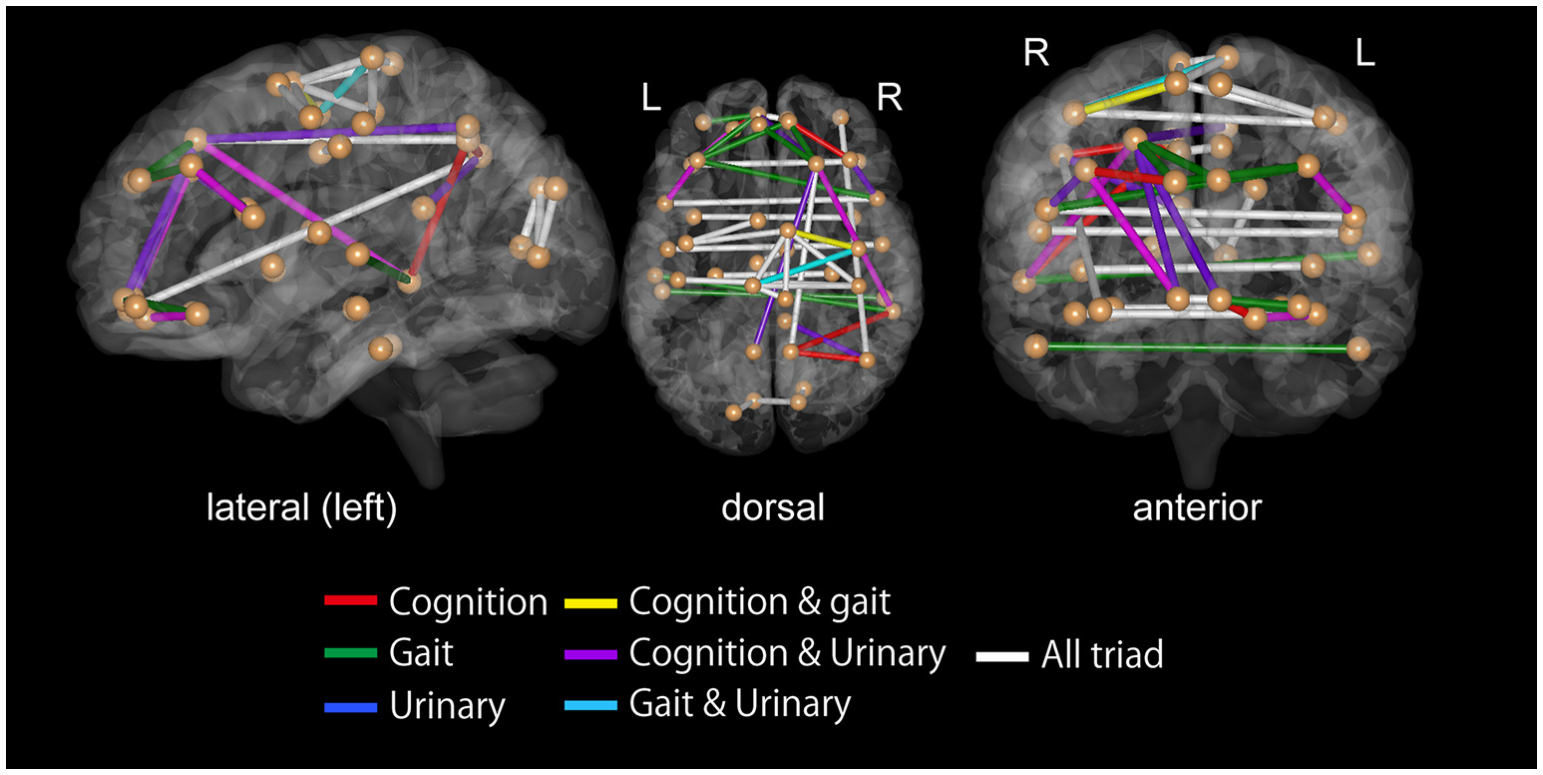
Top 30 functional connectivity with higher SVM weights in classification for iNPH grading scale as viewed from the left **(left)**, top **(middle)**, and front **(right)**. Spheres represent the barycenter of each ROI, and lines between spheres stand for connections that showed higher SVM weights listed in Tables 3–5.

For all the three symptoms, the assessment of SVM weights indicated the importance of inter-hemispheric functional connectivity. The top 30 FCs with high contributions to classification are shown in Tables 3–5; Figure 4. The sum of these top 30 weights accounts for 16.9% of the total weights for iNPH-GS gait, 16.4% for iNPH-GS cognition, and 15.0% for iNPH-GS urinary incontinence.

**Table 3:** SVM weight for classification with iNPH grading scale for gait disturbance.

**Functional connectivity**	**Weight of SVM**
*iNPH-GS gait*	
**R postcentral gyrus–L postcentral gyrus**	0.048
**R paracentral lobule–L paracentral lobule**	0.047
**R median cingulate gyri–L median cingulate gyri**	0.047
**R superior frontal gyrus, medial orbital–L superior frontal gyrus, medial orbital**	0.046
R cuneus–R calcarine fissure	0.046
R superior frontal gyrus, medial orbital–R superior frontal gyrus, dorsolateral	0.042
**R cuneus–L calcarine fissure**	0.041
L superior occipital gyrus–L calcarine fissure	0.039
R paracentral lobule–R supplementary motor area	0.038
**R inferior frontal gyrus, orbital part–L inferior frontal gyrus, orbital part**	0.036
R supplementary motor area–R precentral gyrus	0.036
**L paracentral lobule–R supplementary motor area**	0.036
**L paracentral lobule–R postcentral gyrus**	0.036
R angular gyrus–R middle frontal gyrus, orbital part	0.035
L supplementary motor area–L precentral gyrus	0.034
**R supplementary motor area–L precentral gyrus**	0.033
**R inferior temporal gyrus–L inferior temporal gyrus**	0.032
R postcentral gyrus–R supplementary motor area	0.032
**L paracentral lobule–R precentral gyrus**	0.031
**L superior frontal gyrus, medial orbital–R superior frontal gyrus, dorsolateral**	0.030
**R rolandic operculum–L rolandic operculum**	0.030
R inferior frontal gyrus, opercular part–R middle frontal gyrus	0.030
**R insula–L insula**	0.030
**R middle temporal gyrus–L superior temporal gyrus**	0.030
R precuneus–R superior frontal gyrus, dorsolateral	0.028
R superior frontal gyrus, medial orbital–R middle frontal gyrus	0.028
**R hippocampus–L hippocampus**	0.028
L middle frontal gyrus, orbital part–L superior frontal gyrus, orbital part	0.028
**R inferior frontal gyrus, opercular part–L inferior frontal gyrus, opercular part**	0.028
L inferior frontal gyrus, orbital part–L superior frontal gyrus, orbital part	0.028

*Bold fonts indicate interhemispheric connectivity*.

**Table 4:** SVM weight for classification with iNPH grading scale for cognitive impairment.

**Functional connectivity**	**Weight of SVM**
*iNPH-GS cognition*	
**R superior frontal gyrus, medial orbital**–**L superior frontal gyrus, medial orbital**	0.053
R superior frontal gyrus, medial orbital–R superior frontal gyrus, dorsolateral	0.050
**R median cingulate gyri**–**L median cingulate gyri**	0.049
**R postcentral gyrus**–**L postcentral gyrus**	0.047
**R paracentral lobule**–**L paracentral lobule**	0.046
R cuneus–R calcarine fissure	0.045
**R cuneus**–**L calcarine fissure**	0.041
**R insular cortex**–**L insular cortex**	0.041
L superior occipital gyrus–L calcarine fissure	0.038
L supplementary motor area–L precentral gyrus	0.038
R supplementary motor area–R precentral gyrus	0.036
R precuneus–R angular gyrus	0.036
**R inferior frontal gyrus, orbital part**–**L inferior frontal gyrus, orbital part**	0.036
R angular gyrus–R middle frontal gyrus, orbital part	0.035
**L paracentral lobule**–**R supplementary motor area**	0.035
R paracentral lobule–R supplementary motor area	0.034
R angular gyrus–R posterior cingulate gyrus	0.034
**R hippocampus**–**L hippocampus**	0.033
**L paracentral lobule**–**R postcentral gyrus**	0.033
**R rolandic operculum**–**L rolandic operculum**	0.033
R inferior frontal gyrus, opercular part–R middle frontal gyrus	0.033
R superior frontal gyrus, medial orbital–R middle frontal gyrus	0.033
L middle frontal gyrus, orbital part–L superior frontal gyrus, orbital part	0.032
**L superior frontal gyrus, medial orbital**–**R superior frontal gyrus, dorsolateral**	0.032
R precuneus–R superior frontal gyrus, dorsolateral	0.032
R postcentral gyrus–R supplementary motor area	0.032
**R inferior frontal gyrus, opercular part**–**L inferior frontal gyrus, opercular part**	0.031
R middle temporal gyrus–R precuneus	0.031
**R supplementary motor area**–**L precentral gyrus**	0.030
**L precuneus**–**R superior frontal gyrus, dorsolateral**	0.029

*Bold font indicates interhemispheric connectivity*.

**Table 5:** SVM weight for classification with iNPH grading scale for urinary incontinence.

**Functional connectivity**	**Weight of SVM**
*iNPH-GS urinary*	
**R superior frontal gyrus, medial orbital**–**L superior frontal gyrus, medial orbital**	0.047
**R median cingulate gyri**–**L median cingulate gyri**	0.043
R superior frontal gyrus, medial orbita–R superior frontal gyrus, dorsolateral	0.042
**R postcentral gyrus**–**L postcentral gyrus**	0.036
**R paracentral lobule**–**L paracentral lobule**	0.035
R superior frontal gyrus, medial orbital–R middle frontal gyrus	0.034
R cuneus–R calcarine fissure	0.034
**R cuneus**–**L calcarine fissure**	0.034
**R hippocampus**–**L hippocampus**	0.032
**R insular cortex**–**L insular cortex**	0.032
**R inferior frontal gyrus, orbital part**–**L inferior frontal gyrus, orbital part**	0.032
R precuneus–R superior frontal gyrus, dorsolateral	0.032
R inferior frontal gyrus, opercular part–R middle frontal gyrus	0.031
**L superior frontal gyrus, medial orbital**–**R superior frontal gyrus, dorsolateral**	0.031
L supplementary motor area–L precentral gyrus	0.030
R angular gyrus–R middle frontal gyrus, orbital part	0.030
**L precuneus**–**R superior frontal gyrus, dorsolateral**	0.029
R supplementary motor area–R precentral gyrus	0.029
L superior occipital gyrus–L calcarine fissure	0.028
**R inferior frontal gyrus, opercular part**–**L inferior frontal gyrus, opercular part**	0.028
**L paracentral lobule**–**R supplementary motor area**	0.028
L inferior frontal gyrus, orbital part–L superior frontal gyrus, orbital part	0.028
L inferior frontal gyrus, opercular part–L middle frontal gyrus	0.028
**L paracentral lobule**–**R postcentral gyrus**	0.027
R middle temporal gyrus–R superior frontal gyrus, dorsolateral	0.027
**R rolandic operculum**–**L rolandic operculum**	0.027
R angular gyrus–R posterior cingulate gyrus	0.027
**L paracentral lobule**–**R precentral gyrus**	0.026
R postcentral gyrus–R supplementary motor area	0.026
**L postcentral gyrus**–**R supplementary motor area**	0.026

*Bold font indicates interhemispheric connectivity*.

To confirm the significance of inter-hemispheric FC for classification, we calculated the sum of weights of inter-hemispheric FC and that of intra-hemispheric FC, all of which contributed to the prediction of iNPH-GS (Table 6).

**Table 6:** Sum of SVM weights from inter-hemispheric and intra-hemispheric connectivity contributing to prediction of iNPH-GS.

	**iNPH-GS gait**	**iNPH-GS cognition**	**iNPH-GS urinary**
Inter-hemispheric connectivity	3.361	3.568	4.057
Intra-hemispheric connectivity	2.873	3.190	3.441

The results showed greater contribution of the inter-hemispheric FC than that of the intra-hemispheric FC significantly for gait (*p* = 0.02, Wilcoxon rank-sum test), cognition (*p* = 0.002), and marginally for urinary symptoms (*p* = 0.07).

## Discussion

The SVM classifier, combined with feature selection, was able to learn differences in high-dimensional FCs between the iNPH patients, and the healthy controls, thereby successfully classifying each participant with an accuracy of 80%. Considering the importance, and difficulty of a clinical diagnosis of iNPH, rsfcMRI is promising as a tool for providing a biomarker for the diagnosis of iNPH. Furthermore, the assessment of the spatial distribution of FC contributing to classification has shed light on the pathophysiology of iNPH.

FCs having relevance to the classification of iNPH included the superior temporal pole, middle temporal pole, insula, orbitofrontal cortex, and medial frontal cortex. Almost all of these areas were located near the enlarged cerebral sulci (i.e., increased CSF space) in iNPH patients as shown in a previous voxel-based morphometry study (Yamashita et al., 2010, 2014). In a study by Lenfeldt et al. (2008), the left dorsal premotor cortex and bilateral supplementary motor areas (SMA) showed enhanced activation during hand motor task performance after a three-day continuous CSF drainage in iNPH patients (Lenfeldt et al., 2008). In the present study, FC between insula and SMA, and between bilateral postcentral gyri showed relatively high contribution to the classification of iNPH, and HC as indexed by the SVM weights. Additionally, it is suggested that that the effectiveness of CSF drainage possibly results from the reversal of subcortical chronic ischemia, especially in the periventricular zones (Momjian et al., 2004; Malm and Eklund, 2006; Lenfeldt et al., 2008). Our results suggest that the contribution of periventricular areas to the iNPH-HC classification may indicate the abnormality of these FCs because of the compression of fiber connections by enlarged ventricles.

Moreover, the prediction of iNPH-GS indicated the relevance of inter-hemispheric connectivity to the discrimination of the triad: gait disturbance, cognitive impairment, and urinary incontinence. This finding is consistent with the idea that damage to the inter-hemispheric connections via the corpus callosum may underlie the pathophysiology of iNPH. A majority of previous diffusion tensor imaging studies have observed lower fractional anisotropy (FA) values in the corpus callosum in iNPH patients than in normal controls. For instance, Koyama et al. showed that there was a conspicuous decline in FA values in the corpus callosum in iNPH patients, and that the degree of FA reduction was associated with the severity of gait disturbance, which is the most frequent clinical manifestation of iNPH (Koyama et al., 2012, 2013). Consistent with this result, the reduction of FA in the corpus callosum is correlated with gait disturbance and cognitive decline in patients with age-related white matter changes (Iseki et al., 2015). In this study, the corpus callosum connections are suggested to mediate information relevant to gait planning.

Previous imaging studies suggest that the micturition center is located in the pons, and that SMA is involved in the inhibitory control of the micturition reflex (Fukuyama et al., 1996; Zhang et al., 2005). Because we did not include the pons in the ROI set, the results of the present study would mainly reflect the decline of functional connections to and from the SMA related to the inhibitory control of micturition. The insular cortex and cingulate cortex are thought to be involved in the regulation of the bladder filling sensation and void control (Fowler et al., 2008; de Groat et al., 2015). In our results, the interhemispheric connectivity of cingulate cortex, and insular cortex showed respectively high weights for iNPH-GS urinary classification (see Table 5).

To account for cognitive impairment in iNPH, the present finding of inter-hemispheric disconnectivity in iNPH is consistent with the findings that the severity of Alzheimer's disease is correlated with the reduction of volume and FA in the corpus callosum (Chaim et al., 2007; Tomaiuolo et al., 2007). Additionally, Khoo and coworkers have demonstrated that the reduction of RSFC in the DMN was positively correlated with iNPH-GS cognition and urinary incontinence in the iNPH patients (Khoo et al., 2015). Consistent with this result, the present study showed that interhemispheric connectivity in the medial orbital part of the superior frontal gyrus, a part of the DMN, had high weight for all symptoms for iNPH-GS classification. Several researchers reported that white matter alterations in NPH include not only the reduction of FA, but also the alteration of mean diffusivity (MD) and radial diffusivity (RD) (Hattingen et al., 2010; Kanno et al., 2011). In addition, a recent study by Horinek et al. demonstrated reduced FA in the corpus callosum and increased FA, MD, and RD in the cortico-fugal fibers in NPH patients (Horinek et al., 2016). It is possible that the alteration of interhemispheric FC in the iNPH patients shown here is founded on the structural changes of the white matter indexed by those diffusion measures. In sum, the present study suggests the importance of interhemispheric functional connectivity in accounting for the triad of iNPH. For the treatment of iNPH, it is well-recognized that the symptoms are relievable by a shunt operation. The improvement of iNPH triads by CSF drainage can be explained by the idea that a shunt operation releases the compression of the corpus callosum and restores the inter-hemispheric functional connectivity. In a study by Scheel et al. (2012), abnormality of the corpus callosum FA in iNPH patients showed a trend toward normalization following shunt surgery. It is likely that the enlarged ventricles compressed the corpus callosum, affecting inter-hemispheric functional connectivity. The present findings also suggest that the degree of such compression and the resulting functional disconnectivity are correlated with the severity of iNPH symptoms.

This study has several limitations. First, the present data are derived from a relatively small number of participants. Moreover, we acquired data with the same MRI aperture and EPI sequences, but in different hospital sites. This was for a purely practical reason; scanning of the healthy population was not possible in one hospital, and iNPH patients were not available in the other hospital. It is possible that differences in the sites affected the characteristics of the MRI data, because between-site reliability might be relatively low compared with test-retest reliability (Friedman et al., 2008). However, Turner and collages carried out a multi-site rsfcMRI study on schizophrenia patients, and suggested that critical RSFC information important for disease classification was preserved when the scanner model, and scanning parameters were the same. In the present study, we used the same MRI model, and scanning parameters. Therefore, we believe that the difference in site did not critically impair the RSFC information important for the disease/severity classification.

Second, we were not able to associate the present findings with the information regarding treatment of iNPH, such as responsiveness to a CSF tap test. Aoki et al. (2013, 2015) found that responders to a shunt operation showed higher variance in the power of beta-frequency electroencephalography oscillations in the right fronto-temporo-occipital region than non-responders. Because a CSF tapping test is an invasive procedure and carries some risks of adverse events, we agree that predicting the responsiveness to a CSF tap test beforehand is of clinical benefit. However, we only had two negative responders to a tap test, precluding further analysis between responders, and non-responders. The question of whether rsfcMRI-derived functional connectivity can predict the responsiveness to CSF drainage should be tested in the future.

Finally, it may be possible to enhance classification accuracy by improving the machine-learning algorithm. Recently, many methods have been proposed such as linear discrimination analysis, random forest, and sparse logistic regression (Yamashita et al., 2008). We used SVM for the machine-learning classifier, because SVM does not need many samples under the condition that the number of feature dimensions is optimally reduced (Hua et al., 2005). Moreover, classification accuracy may be further improved by optimizing the cost function, kernel type, and classification algorithm. However, considering the computational cost, the exploration of the huge parameter space is beyond the scope of the present study. Future challenges include the improvement of classification accuracy by optimizing the classifiers and feature selection methods. Future challenges include the improvement of classification accuracy by optimizing the classifiers and feature selection methods.

## Conclusion

In conclusion, a machine-learning algorithm combined with rsfcMRI data was able to discriminate between patients with iNPH and elderly controls. This result indicates that iNPH patients have abnormalities in the RSFC, especially in inter-hemispheric functional connectivity, which contributed to the classification of clinical stages. RsfcMRI may have the potential to discriminate iNPH from other neurological diseases, contributing to a differential diagnosis of iNPH.

## Author contributions

YOg designed the study, performed analyses and literature review, and drafted the manuscript. AO, MO, NN, HT, NS recruited healthy volunteers and iNPH patients and performed experiments. YOk developed experimental and analytic programs. TH designed the study and supervised the research and revised the manuscript. All the authors read and approved the final manuscript.

### Conflict of interest statement

The authors declare that the research was conducted in the absence of any commercial or financial relationships that could be construed as a potential conflict of interest.
